# A Communication-Based Intervention Study for Reducing Stigma and Discrimination against Tuberculosis among Thai High-School Students

**DOI:** 10.3390/ijerph20054136

**Published:** 2023-02-25

**Authors:** Saowaluk Moonsarn, Yuthichai Kasetjaroen, Anne-Marie Bettex-Baars, Anuchit Phanumartwiwath

**Affiliations:** 1 College of Public Health Sciences, Chulalongkorn University, Bangkok 10330, Thailand; 2 Division Control of AIDS, TB and STI, Bangkok Metropolitan Administration, Bangkok 10600, Thailand; 3 BE Health Association, 1204 Geneva, Switzerland

**Keywords:** tuberculosis, stigma, discrimination, communication intervention, high-school students

## Abstract

The current study aims to explore the effectiveness of communication-based intervention on the reduction in TB stigma and discrimination among high-school students in Bangkok, Thailand, during the COVID-19 outbreak. This study is quasi-experimental in nature and is conducted in two high schools (*n* = 216 students). The study adopts purposive and systematic sampling techniques to select schools and students. The experimental group received a communication program for three months, whereas the control group received no intervention. The study uses generalized estimating equations to assess the overall program between the experimental and control groups at baseline, intervention, and follow-up periods. The outcomes reveal that the communication program effectively reduced TB stigma (*p*-value < 0.05, CI = 4.962, −1.723) and increased knowledge about TB (*p*-value < 0.05, CI = 1.825, 2.537), attitudes toward TB (*p*-value < 0.05, CI = 4.493, 6.280), and self-efficacy on TB stigma and discrimination (*p*-value < 0.05, CI = 7.133, 9.483) compared with the control group. However, the study finds no significant within- and between-group differences in TB discrimination (*p*-value > 0.05, CI = −1.398, 0.810). This study is applicable as a supplement for knowledge and attitudes about TB and to the reduction in TB stigma in schools.

## 1. Introduction

Thailand is a country with a high rate of tuberculosis (TB) and infection TB/HIV [1]. In 2020, Thailand reached an estimated 150 cases per 100,000 of the population. In addition, 17,000 of those patients had no access to medical services and treatments [2]. At the same time, an estimated 11,833 TB cases occurred in Bangkok, of which 800 people remained without treatment [3]. This partly reflects a delay in or lack of access to treatment, leading to a spread of the disease in these communities. However, poverty, education and other social risk factors, of which stigma is one component [4].

Stigma is associated with tuberculosis (TB) diagnosis; the main causes of stigma are myths about transmission, association with poverty, lack of knowledge [5], and association with HIV/AIDS because so many people with HIV/AIDS die of TB [6]. Therefore, the stigma is highly associated with TB. Stigma is a social process that exists when elements of labeling, stereotyping, separation, loss of status, and discrimination occur in an enabling power situation [7]. Stigma originates from the Greek language, meaning a mark or brand. Goffman (1963) describes it as a social reality where a person is identified by a specific attribute, behavior, or reputation considered undesirable or discrediting, which leads to being negatively regarded by society and, therefore, devalued or even rejected [8]. Stigma is a result of prejudice, although people with good education continue to fear being infected with TB [5]. However, the biggest problem with stigma is that patients with TB, in particular, the younger population, feel unworthy or guilty and tend to stigmatize themselves [5]. Such internalized stigma leads to depression and self-isolation, which potentially drives withdrawal from TB treatment. External stigma or the actual social experience of discrimination, increases along with internal or internalized stigma. Thus, TB stigma is one of the major social factors that triggers a delay in diagnosis and non-adherence to treatment among patients with TB [9]. TB stigma and discrimination exert an impact on whether or not one suffers at home, in the workplace/institution, or in the community. Furthermore, several studies report that TB stigma also occurs in nurseries, schools, hospitals, workplaces, and other institutional settings [10]. The results of Osonwa and Eko [11] revealed that 83.3% of participants ceased purchasing food from a person with TB, 36.0% avoided crowded areas, and 31.5% avoided eating, talking, and sharing a bed with a patient with TB. Hence, previous studies report high rates of social stigma and discrimination against patients with TB among school adolescents in Ogoja, a local government area in Cross River State, Nigeria. Similarly, in 2020, the BE Health Association [12] conducted a survey on four high schools in Bangkok and reported that 57.1% of 630 students acknowledged high levels of TB stigma and discrimination. In addition, the study conducted by Mokhtar et al. [13] showed that 40.5% of participants felt uncomfortable when they sat near TB patients, 34% stated that they felt afraid of TB patients and 29% avoided any physical contact with TB patients. The level of social stigma was reported to be high among adolescents in Penang, Malaysia. Therefore, these numbers should be considered, because a report by the Bangkok Metropolitan Administration (BMA) on TB in 2019 revealed that nearly 5% of patients with TB comprised an estimated 250 new cases of TB among adolescents aged 15–19 years [3]. Few reports conducted by Osonwa and Eko, BE Health Association, Mokhtar et al., and the BMA [3,11,12,13] imply that TB stigma and discrimination are strongly associated with high-school students because the school is a place where TB stigma and discrimination can easily erupt. Several studies have shown many interventions which aim to reduce TB stigma and discrimination [14] by focusing on the general community [15], TB patients [16], healthcare workers [6] as well as adolescents [17]. However, these interventions have certain limitations.

To address this concern, the study aims to explore the effectiveness of communication-based interventions for reducing TB stigma and discrimination and improve TB knowledge and attitudes as well as self-efficacy among high-school students in Bangkok.

Furthermore, the results may benefit students affected by TB in schools and individuals in other institutions, and may facilitate the reduction in TB stigma and discrimination.

## 2. Materials and Methods

### 2.1. Design, Population, and Setting

This study was quasi-experimental in nature and used two groups with a pre- and post-test design. The study was conducted from July to November 2021, and the population consisted of Thai male high-school students in male public high schools located in Bangkok. We selected male high-school students, because the TB cases reported in Bangkok in 2020 comprised an estimated 678 new cases of TB found in adolescents aged 15–24 years. Similarly, an estimated 459 new cases of TB in adolescents aged 15–19 years were found; 57% of TB cases were in men, whereas for the remainder, 33% of TB cases in women [3]. Furthermore, the report of BE Health Association (2020), it was found that there were 57.1% of the 630 high-school students with a high level of TB stigma and discrimination among high-school students in Bangkok. In addition, most male high-school students (25.6%) perceived TB stigma and discrimination at higher levels than female students (21.7%) [12]. Therefore, the implementation area was in male public high-schools located in Bangkok.

### 2.2. Sampling

A purposive sampling was used to select the schools; the criteria included a high incidence of TB cases, a large size (a minimum of approximately 2500 students in total), and location where was the place surrounded by slum areas. The study setting included two male public high schools in Bangkok designated as Schools A (experimental group) and B (control group). The sample size was calculated via comparisons of two mean scores for stigma according to Moya [7,18], and the use of a formula developed by Lemeshow (1990) [19]. The minimum sample size needed for each group of 90 participants. However, the current study anticipated a withdrawal of participants at approximately 20%. The study used the systematic sampling technique to select students with at least three participants per room in each grade level. The number of students per room was at least 40 subjects, and the participants were selected to use 13-digit student identification (ID) numbers by Microsoft Excel. There were 12 rooms per grade level, and the total of classes were 36, with grades between 10 and 12. The total number of participants is 216, which were randomly and equally assigned into two groups, namely, the experimental and control groups.

### 2.3. Research Criteria

The participants consisted of male Thai high-school students (15–19 years) in Grades 10–12 at public high schools in Bangkok. They must be able to read, write, and communicate in the Thai language and use the Line application on social media devices (e.g., smartphones, computers, iPads, tablets, and laptop). The students provided informed consent and expressed voluntary commitment to cooperate. Those who missed the program for two out of 12 sessions, moved to other schools during the semester, and were prohibited by their parents to participant were excluded.

### 2.4. Intervention

The study designed a communication-based intervention based on self-efficacy theory and social support developed by Albert Bandura (1977) [20] and House (1981) [21], respectively. The intervention was provided during this five-month study, implemented within three months, and followed-up and monitored during a two-month program evaluation. The communication-based intervention consisted of four modules, namely, module 1 (TB prevention and care training; once for 2 h per week for weeks 1–3); module 2 (automatic thought in relation to the received stigma training; once for 2 h per week for weeks 4–6); module 3 (building perceptions, confidence, and encouragement to reduce TB stigma and discrimination; once for 2 h per week for weeks 7–9), and module 4 (information support through videos and messages; once for 2 h per week for weeks 10–12). The content of the communication-based intervention was adapted from the curricula for TB and Care Prevention Guidelines [22] and HIV/AIDS stigma and discrimination in healthcare centers [23] and from a self-stigma reduction program [24]. In addition, three experts in the field of pulmonary TB reviewed the communication-based intervention and approved the content and method outlined for the target group. The intervention was conducted by the research team and experts in the field of the TB such as physician and public health professional during the COVID-19 pandemic via an online training using the abovementioned social media channel and devices. The experimental group received the communication-based intervention for three months, whereas the control group did not receive that intervention.

### 2.5. Measurement Tools and Data Collection

Data were collected via questionnaires. The following independent variables were included: socio-demographics (e.g., age, classes, reside with, area of residence, type of accommodation, marital status of parents, level of education of mothers, level of education of fathers, family income and experiences with illness) and information related to TB. Knowledge about and attitudes toward TB and self-efficacy on TB stigma and discrimination were the intermediate outcome variables, whereas TB stigma and discrimination were the main outcome variables. The questionnaire on TB knowledge was modified and developed on the basis of the TB and Care Prevention Guidelines [22] as well as of the study of Pengpid (2016) [25]. The study used a total of 15 questions to assess the overall knowledge of students about TP. Incorrect and correct responses took values of 0 and 1, respectively. The total scores for the knowledge of students were classified into three levels using Bloom’s cut-off point [26], namely, 0–8 points (<60%; poor), 9–12 points (60–80%; moderate), and 13–15 points (>80%; high). The questionnaire for TB attitude was modified and developed using previous studies [11] and the TB Stigma Measurement Guidance [27]. In total, 10 questions were rated using a five-point Likert-type scale: 5 = strongly agree, 4 = agree, 3 = don’t know, 2 = disagree, and 1 = strongly disagree. Scores ranged from 10 to 55. The questionnaire on self-efficacy on TB stigma and discrimination was the participants’ confidence to carry out and reduce the negative behaviors or actions that toward TB patients. The questionnaire was modified and developed from Li et al. (2011) [28] and Johnson et al. (2007) [29]. In total, 14 items were rated using a five-point Likert-type scale: 5 = strongly confident, 4 = very confident, 3 = partially confident, 2 = unsure, and 1 = not confident. Scores ranged from 14 to 70. The questionnaire for TB stigma was modified and developed using the TB stigma Measurement Guidance [27]. Items were rated using a five-point Likert-type scale: 4 = strongly agree, 3 = agree, 2 = don’t know, 1 = disagree, and 0 = strongly disagree. Scores ranged from 0 to 44. The questionnaire on TB discrimination was modified and developed using the TB stigma Measurement Guidance [27]. Items were rated using a five-point Likert-type scale: 4 = strongly agree, 3 = agree, 2 = don’t know, 1 = disagree, and 0 = strongly disagree. Scores ranged from 0 to 40.

### 2.6. Validity and Reliability Tests

Three experts in the field of pulmonary TB conducted the validity test of the questionnaire, which was revised according to their comments. The results of the reliability test of the questionnaires consisted of the Kuder–Richardson 20 (KR-20) formula for TB knowledge (0.876), for TB attitudes (0.875), for self-efficacy on TB stigma and discrimination (0.744), TB stigma (0.907), and TB discrimination (0.825).

### 2.7. Analysis

Data were analyzed using SPSS for Windows version 20 (IBM Statistics, Armonk, NY, USA). Descriptive statistics comprised the chi-squared test, Fisher’s exact test, and independent sample *t*-test to analyze the socio-demographic factors at baseline. The Kolmogorov–Smirnov test was used to assess the normality of variables. The independent sample *t*-test was used to compare between the experimental and control groups, whereas a paired *t*-test was used for a comparison of differences in mean scores within the same group. The study also employed generalized estimating equations (GEE) to test the overall program between the experimental and control groups at baseline, three-month intervention and two-month follow-up. GEE was also used to eliminate any potential factors at baseline in the groups that may impact the findings of the study. Statistical significance was set to a *p*-value less than 0.05.

### 2.8. Ethics Approval

Ethical approval for the study was obtained from the Research Ethics Review Committee of Chulalongkorn University, Thailand (approval number 054.1/64). Letters were written to the principals of the high schools to obtain permission and cooperation for data collection. Informed consent was secured prior to the study. Students aged 18 years or older provided informed consent on a voluntary basis, whereas students aged less than 18 years provided informed consents on a voluntary basis with the approval of their parent or guardian.

## 3. Results

### 3.1. Socio-Demographic Factors

The study recruited a total of 216 students, whereas 4 students who were excluded from the control group did not meet the inclusion criteria and refused to participate in this study. After signing the informed consent, the remaining 212 students were selected to be assigned as the experimental (108 students) and the control groups (104 students). The dropout rate was expected to be <20% after additional recruitment. Table 1 provides a summary of the baseline socio-demographic characteristics of the participants and indicates statistically significant differences between the two groups in terms of the level of education of mothers, level of education of fathers, and family income (*p*-values < 0.05), whereas the other baseline factors differ significantly between them (*p*-value > 0.05).

### 3.2. Results of between-Group Comparison and Differences in Mean Scores

Table 2 provides the results of comparisons between the two groups. At baseline, data demonstrated no statistically significant differences in TB attitudes, self-efficacy on TB stigma and discrimination, TB stigma and discrimination (*p*-value > 0.05), but the study observed a statistically significant difference in TB knowledge (*p*-value = 0.022). In contrast, after three months of implementation, the outcomes revealed statistically significant differences in TB knowledge, TB attitudes, self-efficacy on TB stigma and discrimination, and TB stigma (*p*-value < 0.05) but not for TB discrimination (*p*-value > 0.05). The study collected the same results after five months.

### 3.3. Results of within-Group Comparison and Differences in Mean Scores

Table 3 presents the results of within-group comparison. TB knowledge, TB attitudes, and self-efficacy on TB stigma and discrimination improved across the three time periods (*p*-value < 0.05). Although the reduction in TB stigma was only improved from baseline to month three and from months three to five (*p*-value < 0.05). Moreover, the study observed no significant difference in the reduction in overall TB discrimination across three time periods in the experimental group (*p*-value > 0.05). In contrast with the control group, TB attitudes and the reduction in TB discrimination were not improved across the three time periods (*p*-value > 0.05). TB knowledge was only improved from baseline to month three and from months three to five (*p*-value < 0.05). Similarly, the reduction in TB stigma displayed a significant increase from baseline to month five and from months three to five (*p*-value < 0.05). However, self-efficacy on TB stigma and discrimination exhibited a significant increase from baseline to month five for the control group (*p*-value = 0.024).

### 3.4. Testing the Effectiveness of the Intervention on Reduction in TB Stigma and TB Discrimination

Table 4 presents the comparisons of the overall program for TB knowledge, TB attitude, self-efficacy on TB stigma and discrimination, and TB discrimination between the groups. Afterward, the study adjusted for the level of education of mothers, level of education of fathers, and family income to test the effectiveness of communication on TB knowledge, TB attitude, and self-efficacy on TB stigma and discrimination, and the reduction in TB stigma and discrimination. Compared with the control group, the results revealed that the communication program was effective in reducing TB stigma (*p*-value < 0.05, CI = 4.962, −1.723) and increasing TB knowledge (*p*-value < 0.05, CI = 1.825, 2.537), TB attitude (*p*-value < 0.05, CI = 4.493, 6.280), and self-efficacy on TB stigma and discrimination (*p*-value < 0.05, CI = 7.133, 9.48). However, the study found no statistically significant difference for TB discrimination within and between the two groups (*p*-value > 0.05, CI = −1.398, 0.810).

## 4. Discussion

This study used the GEE analysis to adjust potential factors at the baseline such as mother’s education status, father’s education status, and family income in the groups. After adjusting the potential factors, the results revealed that these measures demonstrated the significant effectiveness of communication-based intervention in reducing TB stigma for the experimental group, whereas TB discrimination did not exhibit a significant change within and between both groups. The study found that the intervention exerted an effect on TB stigma, which significantly reduced internalized stigma in patients with TB after two months [18]. In addition, a community-based intervention significantly reduced anticipated stigma in the general community [15]. However, the study found no significant intervention effect on TB discrimination between the experiment and control groups. Moreover, discriminatory attitudes and isolated behaviors toward TB did not change significantly in southeastern China [30]. Furthermore, the majority of the participants seemingly never met persons with TB, which indicates that none of them gained the opportunity to witness or experience TB discrimination. Hence, TB discrimination was more prevalent in other groups than that among high-school students, such as patients with TB, persons affected by TB, and without treatment [31]. In addition, the results demonstrated that a communication-based intervention can exert a positive effect on the improvement of TB knowledge, TB attitude, and self-efficacy on TB stigma and discrimination within the experimental group and between groups. These findings are similar to the report of Bisallah et al. (2018) [32], which indicated that health education intervention resulted in significant improvement in TB knowledge and TB attitude within the experimental group compared with the control group. Furthermore, we found that the intervention, which was based on self-efficacy theory, demonstrated that TB infection control was improved through TB knowledge and TB attitude after the intervention [33].

Therefore, the results indicated that a communication-based intervention based on self-efficacy and social support theories were effective in reducing TB stigma and improving TB knowledge, TB attitude, and self-efficacy on TB stigma and discrimination compared with the control group. The findings suggested that the participants who completed this intervention felt confident to convey anti-TB stigma messages. In addition, the participating students, who felt self-assured in communicating what they learned to people in their immediate surroundings, were aware of the social effects exerted by TB on the community, and gained prevention behavior with regard to pulmonary TB infection. Furthermore, we recommend that future studies should further investigate the questions and lessons generated by the intervention, which could add value to potential identical interventions to increase TB knowledge, TB attitude, and self-efficacy on TB stigma and discrimination and to reduce TB stigma in schools and other institutions. In addition, a further investigation of this study should increase the number of subjects enrolled, using a randomization in the assignment to groups, and the number months of training for monitoring a change of attitudes towards TB discrimination.

This study has its limitations. First, this study was quasi-experimental in nature. Therefore, the population could not be randomly selected, because the area and sample size were limited, narrowing the generalization of the study at the same time. Second, this study was conducted via online training due to COVID-19 restrictions. Therefore, the intervention may have been perceived as incomplete for participants who only had access to social media. In addition, the same COVID-19 restrictions prevented the monitoring of the feelings of the participants (emotions during the five-month study period).

## 5. Conclusions

The communication intervention based on self-efficacy and social support theories demonstrated that TB stigma and TB discrimination can be effectively reduced, whereas TB knowledge and TB attitude and self-efficacy on TB stigma and discrimination among high-school students can be increased. Therefore, we recommend that the inclusion of our developed modules for TB stigma reduction into school programs for adolescents, other institutions, and other countries is of the same socio-cultural importance as in Thailand. Furthermore, future interventions to reduce TB stigma should be concerned on TB stigma and the intersection between the socio-cultural aspect and structural determinants of health. 

## Figures and Tables

**Table 1:** Baseline socio-demographic characteristics of the participants. (*n* = 212).

Socio-Demographic Characteristics	Experimental Group (*n* = 108)	Control Group (*n* = 104)	*p*-Value
Age in years, mean (SD)	16.28 (0.91)	16.19 (1.03)	0.524 ^c^
Classes (n, %)			0.981 ^a^
Grade 10	36 (33.3)	36 (34.6)	
Grade 11	36 (33.3)	34 (32.7)	
Grade 12	36 (33.3)	34 (32.7)	
Residing with (n, %)			0.056 ^a^
Both parents	74 (68.5)	87 (83.7)	
Single parents	12 (11.1)	8 (7.7)	
Guardian	21 (19.4)	9 (8.7)	
Living alone	1 (0.9)	0 (0)	
Area of residence (n, %)			0.412 ^a^
Village	48 (44.4)	53 (51.0)	
Commercial buildings	47 (43.5)	36 (34.6)	
Others, specify (slum area or overcrowded area)	13 (12.0)	15 (14.4)	
Type of accommodation (n, %)			0.136 ^a^
Owner house	39 (36.1)	51 (49.0)	
Flats/Apartment/Condo/Commercial buildings	60 (55.6)	44 (42.3)	
Others, specify (slum area or overcrowded area)	9 (8.3)	9 (8.7)	
Parents marital status (n, %)			0.053 ^a^
Married/co-habited	77 (71.3)	88 (84.6)	
Divorced/separated	27 (25.0)	15 (14.4)	
Widowed	4 (3.7)	1 (1.0)	
Mother’s educational status (n, %)			<0.001 ^a^ *
Primary level or lower	18 (16.7)	6 (5.8)	
Secondary level	39 (36.1)	13 (12.5)	
Diploma level	16 (14.8)	6 (5.8)	
Bachelor’s degree or higher	35 (32.4)	79 (76.0)	
Father’s educational status (n, %)			<0.001 ^a^ *
Primary level or lower	28 (25.9)	5 (5.8)	
Secondary level	39 (36.1)	9 (8.7)	
Diploma level	15 (13.9)	11 (10.6)	
Bachelor’s degree or higher	26 (24.1)	78 (75.0)	
Family income per month (n, %)			<0.001 ^a^ *
<15,000 Baht (<USD 445.7)	38 (35.2)	7 (6.7)	
15,000–45,000 Baht (USD 445.7–1342.6)	57 (52.8)	49 (47.1)	
>45,000 Baht (>USD 1342.6)	13 (12.0)	48 (46.2)	
Illness history (n, %)			0.328 ^b^
Yes	18 (16.7)	12 (11.5)	
No	90 (83.3)	92 (88.5)	
Information related with TB			
Ever hear/learning of TB information			0.078 ^b^
Yes	103 (95.4)	92 (88.5)	
No	5 (4.6)	12 (11.5)	
Yes	103 (95.4)	92 (88.5)	
No	5 (4.6)	12 (11.5)	
Source of TB information			0.052 ^a^
TV/Radio	23 (21.3)	18 (17.3)	
Social media (i.e., Facebook/Line/Internet/Instagram/Twitter)	41 (38.0)	46 (44.2)	
Healthcare staffs/Teachers/Community Health Volunteers/Community leaders/Friends/Family	33 (30.6)	19 (18.3)	
Others, specify (Newspaper/journals)	11 (10.2)	21 (20.2)	

*p*-value < 0.05 by ^a^. chi-squared test, ^b^. Fisher’s exact test, and ^c^. independent sample *t*-test. * Statistical significance was determined at *p*-value < 0.05.

**Table 2:** Between-group comparisons of knowledge of TB, attitude of TB, self-efficacy on TB stigma and discrimination, TB stigma and TB discrimination at baseline, month 3 and month 5.

Variable	Baseline			3 Months for Intervention			2 Months for Follow-Up		
	Exp.	Control	*p*-Value	Exp.	Control	*p*-Value	Exp.	Control	*p*-Value
Knowledge of TB, mean (SD)	9.63 (1.46)	9.16 (1.54)	0.022 *	12.83 (1.79)	7.59 (1.87)	<0.001 *	13.77 (1.31)	9.43 (3.07)	<0.001 *
Attitude of TB, mean (SD)	34.88 (4.11)	35.39 (5.72)	0.460	42.27 (5.75)	34.44 (4.61)	<0.001 *	44.53 (4.17)	34.26 (4.12)	<0.001 *
Self-efficacy on TB stigma and discrimination, mean (SD)	37.43 (7.22)	38.67 (8.37)	0.250	43.29 (6.43)	36.49 (7.94)	<0.001 *	51.56 (4.10)	36.16 (5.66)	<0.001 *
TB stigma, mean (SD)	22.43 (7.84)	21.65 (9.05)	0.502	17.52 (7.95)	22.50 (8.44)	<0.001 *	21.36 (8.66)	25.97 (7.73)	<0.001 *
TB discrimination, mean (SD)	28.39 (6.00)	29.15 (6.88)	0.395	27.25 (7.66)	29.04 (6.76)	0.072	26.79 (5.75)	28.15 (5.27)	0.075

Between-group comparisons were analyzed using independent sample *t*-test. Significant at *p*-value < 0.05. * Statistical significance was determined at *p*-value < 0.05.

**Table 3:** Comparison results and mean score differences of knowledge of TB, attitude of TB, self-efficacy on TB stigma and discrimination, TB stigma and TB discrimination within the experimental (*n* = 108) and control groups (*n* = 104) at baseline, month 3 and month 5.

Variable (Time Point)	Experimental		Control	
	Mean Difference	*p*-Value	Mean Difference	*p*-Value
Knowledge of TB				
Baseline to month 3	−3.194 *	<0.001 *	1.567 *	<0.001 *
Baseline to month 5	−4.139 *	<0.001 *	−0.269	1.000
Month 3 to month 5	−0.944 *	<0.001 *	−1.837 *	<0.001 *
Attitude of TB				
Baseline to month 3	−7.389 *	<0.001 *	0.952	0.437
Baseline to month 5	−9.648 *	<0.001 *	1.125	0.337
Month 3 to month 5	−2.259 *	0.003 *	0.173	1.000
Self-efficacy on TB stigma and discrimination				
Baseline to month 3	−5.861 *	<0.001 *	2.183	0.106
Baseline to month 5	−14.130 *	<0.001 *	2.510 *	0.024 *
Month 3 to month 5	−8.269 *	<0.001 *	0.327	1.000
TB stigma				
Baseline to month 3	4.907 *	<0.001 *	−0.856	0.426
Baseline to month 5	1.074	1.000	−4.317 *	0.002 *
Month 3 to month 5	−3.833 *	0.008	−3.462 *	0.009 *
TB discrimination				
Baseline to month 3	1.148	0.528	0.106	1.000
Baseline to month 5	1.602	0.113	1.000	0.710
Month 3 to month 5	0.454	1.000	0.894	0.744

Within-group comparisons were analyzed using paired *t*-test. Significant at *p*-value < 0.05. * Statistical significance was determined at *p*-value < 0.05.

**Table 4:** Testing the effectiveness of the intervention on reduction in TB stigma and TB discrimination.

Variable (Time Point)	Experimental Mean (SD)	Control Mean (SD)	Difference in Mean (Exp.-Control)	Group × Time *p*-Value (95% CI)
Knowledge of TB				
Baseline	9.63 (1.46)	9.16 (1.54)	0.47	<0.001 * (1.825, 2.537)
3 months for intervention	12.83 (1.79)	7.59 (1.87)	5.23	
2 months for follow-up	13.77 (1.31)	9.43 (3.07)	4.34	
Attitude of TB				
Baseline	34.88 (4.11)	35.39 (5.72)	−0.50	<0.001 * (4.493, 6.280)
3 months for intervention	42.27 (5.75)	34.44 (4.61)	7.83	
2 months for follow-up	44.53 (4.17)	34.26 (4.12)	10.26	
Self-efficacy on TB stigma and discrimination				
Baseline	37.43 (7.22)	38.67 (8.37)	−1.23	<0.001 * (7.133, 9.483)
3 months for intervention	43.29 (6.43)	36.49 (7.94)	6.80	
2 months for follow-up	51.56 (4.10)	36.16 (5.66)	15.40	
TB stigma				
Baseline	22.43 (7.84)	21.65 (9.05)	0.78	<0.001 * (−4.962, 1.723)
3 months for intervention	17.52 (7.95)	22.50 (8.44)	−4.98	
2 months for follow-up	21.36 (8.66)	25.97 (7.73)	−4.61	
TB discrimination				
Baseline	28.39 (6.00)	29.15 (6.88)	−0.75	0.602 (−1.398, 0.810)
3 months for intervention	27.25 (7.66)	29.04 (6.76)	−1.79	
2 months for follow-up	26.79 (5.75)	28.15 (5.27)	−1.35	

The covariate variables such as mother’s education status, father’s education status, and family income were adjusted by Generalized Estimating Equations (GEE). Comparisons were analyzed using GEE. Significant at *p*-value < 0.05. * Statistical significance was determined at *p*-value < 0.05.

## Data Availability

All data are publicly available as noted in the manuscript text.
